# Measuring troublesomeness of chronic pain by location

**DOI:** 10.1186/1471-2474-7-34

**Published:** 2006-04-05

**Authors:** Suzanne Parsons, Dawn Carnes, Tamar Pincus, Nadine Foster, Alan Breen, Steven Vogel, Martin Underwood

**Affiliations:** 1 Centre for Health Sciences, Institute for Health Sciences Education, Barts and the London, Queen Mary's School of Medicine and Dentistry, London, UK; 2 Department of Psychology, Royal Holloway, University of London, London, UK; 3 Primary Care Sciences, Keele University, Keele, Staffordshire, UK; 4 Institute for Musculoskeletal Research and Clinical Implementation, Anglo-European College of Chiropractic, Bournemouth, UK; 5 The Research Centre, The British School of Osteopathy, London, UK

## Abstract

**Background:**

Current measures of pain assess the relative contribution of pain in different body regions to the overall impact of pain. We developed a series of questions to measure the relative 'troublesomeness' of pain in different body regions (the "troublesomeness grid"). The study aimed to determine whether the "troublesomeness grid" is an appropriate measure to assess the severity of pain in different body regions, allowing the comparative severity of pain in different body regions to be assessed.

**Methods:**

We used data from a pilot for a population survey of pain (N = 205) and from the population survey itself (N = 2504) to assess the 'troublesomeness grid's performance. Specifically, its face and content validity using overall and item non-completion rates; its criterion related validity by exploring the relationship between troublesomeness and standard measures of pain, disability, distress and health utility for the five body regions most commonly affected by chronic pain; and its reliability and reproducibility in a test/re-test study.

**Results:**

The troublesomeness grid appeared to have good face validity as it had good completion rates. It also appeared to have good content validity as the percentage agreement between the grid and the pain manikin was high (over 90%). In terms of criterion related validity, troublesomeness was most strongly correlated with pain intensity and health related quality of life, but less with disability and distress. The test-retest reliability was between 80% and 90% for the majority of body regions examined.

**Conclusion:**

The troublesomeness grid is well completed and appears to be an appropriate tool to assess the comparative severity of pain in different body regions.

## Background

Measuring the health impact of painful disorders is problematic, as it is difficult to objectively measure subjective sensations like pain. Pain's overall impact is made up not only of its severity, but also of its effect on other aspects of people's lives such as perceived disability and self-identity [1]. The health impact of chronic pain can be assessed using generic measures, like the Chronic Pain Grade (CPG) [2,3] or location specific measures like the Roland Morris Disability Questionnaire for low back pain [4]. However, these measures do not allow the health impact of pain in different body regions to be measured and compared, as location specific measures typically assess pain's impact using non comparable means of assessment [4,5]. These measures are also complex to use when assessing patients both in clinical and research situations.

The concept of 'bothersomeness' of symptoms may provide a way to explore the comparative burden of pains in different body regions. The concept of bothersomeness was proposed in 1994 to assess symptom severity amongst patients with asthma [6]. It was developed as a summary of outcomes for specific symptoms in a clinical situation. It may be particularly useful for classifying patients who do not have a clear aetiology for their symptoms, as it is concerned with symptoms and not classifying patients according to disease.

Bothersomeness has been explored by researchers in the USA in relation to sciatica [7], and in relation to back pain in the UK [8]. Dunn et al found that bothersomeness was a valid measure of the severity of pain in a group of primary care patients with low back pain and that it was associated with measures of pain, disability, psychological health and work absence [8]. Therefore they concluded that it could be used as a substitute for longer measures, if it was being used to classify patients with low back pain. The bothersomeness of low back pain question was recommended as part of the internationally agreed package of outcome measures for primary care back pain studies [9] and was anglicised to troublesomeness as one of the outcomes of a large scale RCT of manual therapies for low back pain [10].

Therefore, in terms of pain, the majority of work on the concept of troublesomeness/bothersomeness has been undertaken on low back pain. We propose using the bothersomeness question to study the comparative severity of pain in different body regions, as developing a multi-dimensional instrument to measure the impact of pain in different body regions, in a standardised way, would be impractical for routine use.

**Figure 1:**
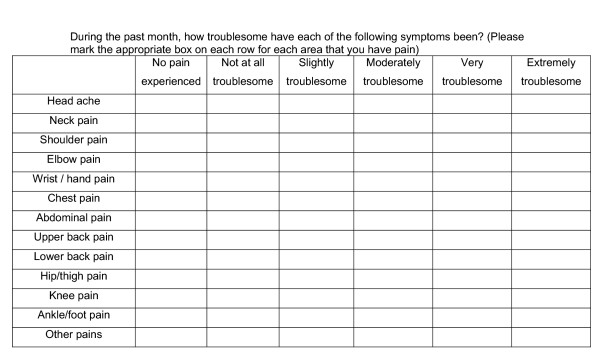
Troublesomeness grid used in the questionnaire.

We used the troublesomeness question to study the comparative impact of pains in 12 body regions. This produced a grid with 12 rows and six columns (Figure 1). We describe here our assessment of the face, content and criterion-related validity of this grid, and also its test-retest reliability for use as a postal questionnaire. The aim of this study is to determine if the "troublesomeness grid" is an appropriate tool to assess the comparative severity of pain in different body regions within a primary care setting. The objectives of the study are to a) examine the face and content validity of the grid; b) investigate its criterion related validity by assessing the relationship between selected items and pain intensity and disability (CPG) [2,3], psychological distress (GHQ 12)[11] and health utility (EQ 5D)[12] and c) test its test-retest reliability over a one month period.

## Methods

### Location of troublesome pain

The focus of our research is on axial musculoskeletal pain and exploring patient's perceptions of how troublesome their pain is in those areas. We selected five regions to allow us to collect data on the troublesomeness of axial musculoskeletal pain (neck, shoulders, upper back, lower back and hip/thigh). Other musculoskeletal regions were added to allow pain in the axial region to be compared to other musculoskeletal pains, e.g. elbow, wrist, knee and ankle/foot. We also included headache, chest and abdominal pain, as these are pains which commonly occur without a distinct aetiology and allow the impact of our selected and other pains to be compared. This approach meant that most painful body regions were covered. These twelve questions were broadly ordered from the top to the bottom of the body (Figure 1). Participants were asked to rate how troublesome pain in these regions had been over the preceding four weeks. We selected a four week preceding period to reduce recall bias and to ensure that we were not measuring just acute problems. A final row in the grid allowed participants to include any other painful regions. Respondents were also given the option to indicate that they did not have pain in any of the areas marked.

### Study samples

#### a) Pilot cross-sectional survey (face, content and criterion related validity)

We did a pilot study for a survey about chronic pain in one practice from the Medical Research Council's General Practice Research Framework (MRC GPRF)[13]. We identified a random sample of 330 patients aged 18 or over from their practice register. Patients were excluded if they had a terminal illness, severe psychiatric disorder or a recognised diagnosis for their pain. The practice sent out the questionnaire with two reminders after two and four weeks, the second by recorded delivery.

#### b) Main cross-sectional survey (criterion and construct related validity)

The main study took place in 16 MRC GPRF practices [13]. It ran in a similar manner to the pilot study. The pilot study did not provide sufficient data to assess the extent to which troublesomeness is an independent construct; and so we used the main study data for this analysis.

#### c) Pilot follow-up survey (reliability)

We piloted our follow-up study in one GPRF practice. A sample of responders to the pilot follow up questionnaire, were sent the questionnaire again one month later to assess the test-retest reliability of the questionnaire items.

Ethical review for this study was provided by the London Multi-Centre Research Ethics Committee.

### Study questionnaire

In addition to the troublesomeness grid, the questionnaire included measures of

#### a) Overall pain

The Chronic Pain Grade (CPG) is a seven-item instrument which measures overall chronic pain severity in two dimensions, intensity and disability. Both intensity and disability are measured on 0–100 scales. A combination of the intensity and disability scores is used to calculate the chronic pain grade, but for this analysis we used the chronic pain grade's separate pain intensity and disability scales rather than combining them to generate grades [2,3].

#### b) Health utility

The EQ 5D which is a measure of health utility over the preceding four weeks on the day of completion, that produces scores from -0.157 to 1.0, where zero = death and 1.00 = perfect health [12]

#### c) Psychological distress

In the pilot study we used the Modified Somatic Perception Questionnaire [14]. This performed badly and so in the main study we used the General Health Questionnaire 12 (GHQ 12) [11]. This is a measure of mental health over the preceding year that produces scores from 0–36 where zero = no distress and 36 = very psychologically distressed. We have used main study data for these analyses.

#### d) Pain distribution

Participants completed a pain manikin indicating where they had experienced pain for most days in the preceding year. We developed a computer programme that allowed us to score presence of pain in 52 different body regions with high inter- and intra-rater reliability [15].

### Analysis

#### 1) Face and content validity

Face validity is the extent to which a measure appears to measure the concept that it is intended to [16]. If the content of the measure appears irrelevant or inappropriate then this is likely to result in poor cooperation in completing the measure and thus poor response rates [17]. We explored this by examining non-response rates to the troublesomeness grid. We considered non-responders to be those who had indicated that they had pain in other questions within the questionnaire, but who had not completed the troublesomeness grid. We hypothesised that the face validity of the troublesomeness grid would be low to those who chose not to complete it, despite having pain. We defined non-completion of the troublesomeness grid as the absence of troublesome pain on the grid, of any degree, in any body region, in those with current pain lasting for more than three months. Data from an associated qualitative study suggested that this was an appropriate way of examining non-response [15].

Content validity concerns the extent to which a measure adequately measures all facets of a concept [16]. Therefore, we hypothesized that if the troublesomeness grid has good content validity that if pain is marked on the grid then it should also be marked on the manikin, and that responses to the troublesomeness grid would be in strong agreement with markings on the pain manikin. We used the pain manikin responses as our comparator despite the manikin assessing pain over most days in the last year, rather than the four weeks used for the troublesomeness grid, as few people with pain in one body region for most days in the last year will have become completely pain free for the last four weeks. We defined item non-response as absence of any degree of troublesome pain on the grid in those who marked pain as being present in the equivalent region on the manikin. Additionally, we checked the percentage agreement between the manikin and the grid. Finally, we checked for the selected body regions completeness by analysing the response to the free choice 'other' row.

#### 2) Criterion related validity of the troublesomeness grid

Measurement of criterion related validity is concerned with demonstrating the accuracy of a measure by confirming that it relates in a predictable manner to established measures [17]. It was impractical to compare the performance of each row of the grid against an established measure for pain in that location, as identifying and ensuring adequate previous validation of the reference measure would be a major undertaking. Furthermore, including an additional 12 validated outcome measures in an unsolicited postal questionnaire could adversely affect response rates. Therefore, we used three existing measures of overall pain and health status as our comparators (CPG – pain intensity and disability components, EQ 5D and GHQ 12). We explored the relationships between each of these measures and the five most prevalent troublesome pain regions (lower back, knee, neck, shoulder, and hip/thigh). We produced box and whisker plots for the two body regions most commonly affected by pain; lower back and knee with our three comparator measures. We hypothesised that the more troublesome pains were, the lower the EQ 5D score would be, the higher the pain intensity and disability scores and the lower the GHQ 12 score. We considered a reasonable association to be a correlation coefficient of 0.3 or over, as most criterion related validity co-efficients tend to be small. A good carefully chosen measure is not likely to show a correlation of greater than 0.5 with the criterion under study, and in an applied setting, such as this study's, a correlation of 0.3 with the criterion under study is common [17]. By extrapolation we can assume that it would behave in a similar manner for the remaining pains for which we have too few data for the analysis.

We further explored criterion related validity and explored the extent to which troublesomeness is an independent construct by using data from the main questionnaire survey to explore which of our included measures (CPG, GHQ12, EQ 5D) were most strongly related to the presence of troublesome pain in each body region. To assess this we constructed a series of logistic regression models with low back, hip/thigh, knee, neck and shoulder troublesomeness as the dependent variables and our three references measures, age, sex, education and working status as independent variables. We dichotomised the troublesome pain variables as follows; patients who had at least moderately troublesome pain were coded as having troublesome pain in that area, and patients who had pain that was not troublesome or only slightly troublesome, were coded as not having troublesome pain. Age, EQ 5D and GHQ 12 scores, pain intensity and disability were treated as continuous variables. Gender was coded into males and females, working status was coded into working and not working and education was coded into left school aged 16 or less and left school aged 17 or over. This modelling allowed us to estimate the proportion of the total variance in the individual troublesomeness scores explained by these; and thus the extent that troublesomeness could be considered to be an independent construct.

#### 3) The reliability of the troublesomeness questions over a one month period

Reproducibility can be defined as the ability of an instrument to yield the same results on repeated applications provided that the subjects remain relatively unchanged on the domain being examined [17]. We therefore sent participants who had completed the troublesomeness grid in the follow up study a re-test questionnaire after four weeks. We hypothesised that we would obtain consistent results between test and retest. We examined test-retest reliability by calculating both percentage agreement and intra-class correlation coefficients. We calculated both as we were concerned that the high level of negative marking on each item of the troublesomeness grid might give a misleadingly high correlation coefficient, overestimating the levels of marked agreements [18]. For the percentage agreement analysis, we considered agreement between test and re-test scores of 80–90% as high [17]. We defined agreement as either a) no pain, or not at all troublesome pain at test and re-test or, b) scores on the troublesomeness grid at re-test no more than one Likert scale point either side of previous score. We then calculated the percentage agreement between test and retest for the five most prevalent pains. For the intra-class correlation analysis, we considered an intra-class correlation of 0.8–1.0 to represent a high level of agreement. All analyses were done in SPSS Version 11.

## Results

From our original baseline pilot sample of 330, six (2%) were excluded at the request of the practice; 205/324 questionnaires were returned giving a corrected response rate of 63%. Of these 105/205 (51%) had chronic pain using the International Association for the Study of Pain definition which is "*pain which has lasted for three months or longer and currently troubles them either all of the time or on and off" *[19]. The response rate to the main study questionnaire was 60% (2504/4171) of whom 41% had chronic pain and 1979 of whom were included in the multi-variate analysis.

The descriptive statistics for the study's health outcome measures (EQ 5D, GHQ 12 and Chronic Pain Grade's pain intensity and disability) are reported in table one. In all cases, those with chronic pain had lower health related quality of life, greater psychological distress, greater pain intensity and pain related disability.

### 1) Face and content validity

The troublesomeness grid was well completed. Of those with chronic pain 100/105 (95%) completed the troublesomeness grid, 5/105 (5%) completed the 'other' row of the grid (eye 1, bladder 1, groin 2, nose 1). Item non-response rates for the five most commonly affected body regions were; lower back 11/67 (16%), knee 19/68 (26%), neck 13/55 (24%), hip/thigh 13/38 (34%), shoulder 13/38 (34%). If respondents had pain in a particular body region on the whole they marked both the pain manikin and the troublesomeness grid. The percentage agreements were as follows: lower back 192/205 (94%), knee 182/205 (89%), neck 187/205 (94%), hip/thigh 189/205 (92%), and shoulder 190/205 (93%). These data suggest that the troublesomeness grid has satisfactory face and content validity

### 2) Criterion and construct validity

**Figure 2:**
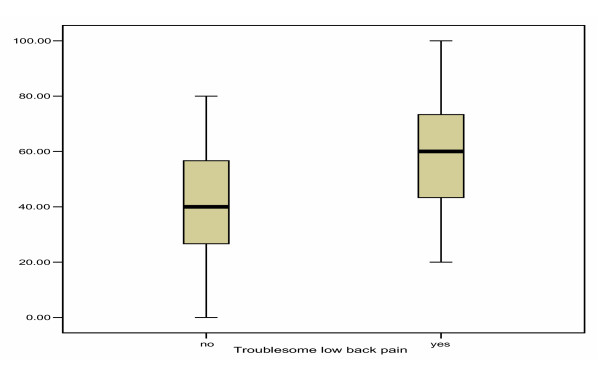
Mean pain intensity scores for individuals with and without troublesome low back pain.

**Figure 3:**
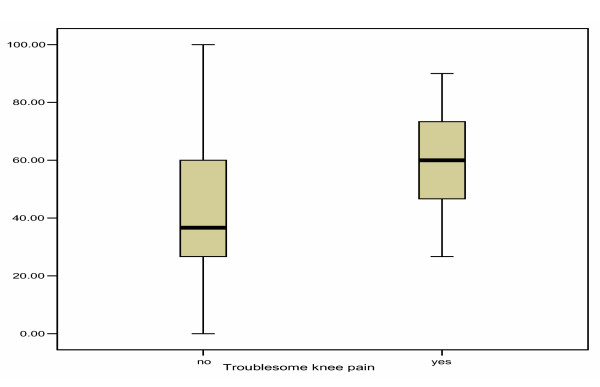
Mean pain intensity scores for individuals with and without troublesome knee pain.

**Figure 4:**
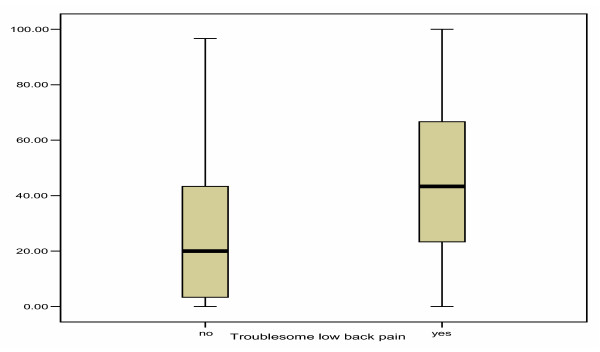
Mean disability scores for individuals with and without troublesome low back pain.

**Figure 5:**
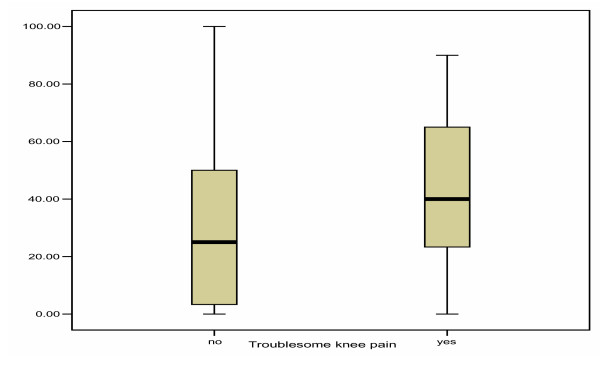
Mean disability scores for individuals with and without troublesome knee pain.

**Figure 6:**
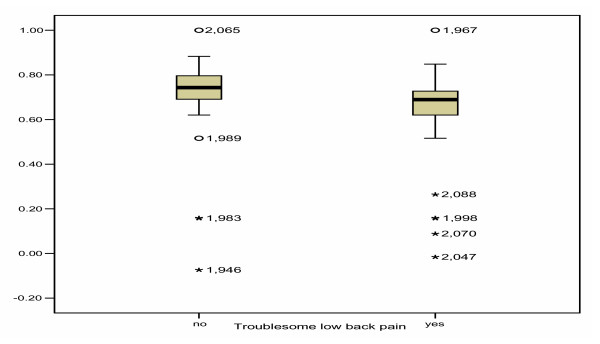
Mean EQ5D scores for individuals with and without troublesome low back pain.

**Figure 7:**
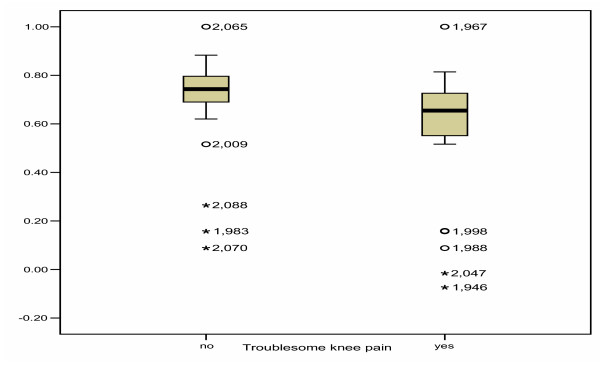
Mean EQ5D scores for individuals with and without troublesome knee pain.

The correlations between individual troublesomeness scores of the five most prevalent troublesome pains (lower back, knee, neck, shoulder and hip/thigh) and the chronic pain grade's pain intensity and disability, the EQ 5D [12], and the GHQ 12 score [11] are summarised in table two. All of the correlations were statistically significant. The correlations with the physical measures (CPG – intensity and disability and EQ 5D) are consistently stronger than those with the GHQ 12, a primarily psychological measure. All of this suggests that troublesomeness may tap into the physical and health related quality of life impact of pain but not into the psychological impact of pain. We calculated the correlation between pain intensity and disability to be 0.84 (p < 0.01), and if we take troublesome low back pain as an example, then pain intensity and pain related disability are more highly related, than troublesome low back pain and pain intensity (0.34; p < 0.002) and troublesome low back pain and pain related disability (0.29; p < 0.01) (Table two). One conclusion from this could be that troublesomeness is measuring a separate construct to pain intensity and disability. Inspection of the box and whisker plots shows that troublesomeness behaves in a predictable manner against each of the comparative measures, i.e. increasing with increasing pain intensity and disability and increasing with decreasing EQ 5D and GHQ 12 scores (Figures 2, 3, 4, 5, 6 and 7).

In the multivariate analyses, pain intensity was a significant explanatory variable for all of the body regions examined, EQ 5D for back, knee and hip, age for knee and hip/thigh and gender for neck, shoulder and hip. In this analysis our independent variables explained 25%–40% of the variance in troublesomeness in each body region (Table three).

### 3) Reliability of the troublesomeness questions over a one month period

Thirty respondents completed the test-retest study. We calculated both the percentage agreement and intra-class correlation coefficients between the test and retest responses to the troublesomeness grid. The percentage agreement according to our definition ranged from 70%–90%. (Table four) The majority of the areas studied where in the acceptable range of 80–90% agreement, apart from agreement between whether troublesome shoulder pain was present at test and retest. Our initial concerns about calculating the intra-class correlation coefficients were not justified as the intra-class correlation coefficients followed the same pattern as the percentage agreement analysis, as all correlation coefficients were between 0.8 and 1.0 apart from shoulder pain.

## Discussion

This study aimed to determine whether the troublesomeness grid was an appropriate measure to assess the burden of pain in different body regions, allowing the comparative burden of pain in different body regions to be assessed. One of the strengths of this study was that we were able to test many aspects of the troublesomeness grid's performance.

### Face and content validity

The troublesomeness grid was well completed, and appeared easy to use and understand. There was a reasonable percentage agreement between the manikin and the troublesomeness grid. Although the overall completion rate was good, the item non-response rates when compared to the pain manikin were higher than we would have liked. This may be partly explained by the difference in time frames used on the two questions, one year versus one month. Although we have used the pain drawing as a 'gold standard' there are few objective data to support the use of pain drawings [15]. Inevitably, there will be some loss of precision in the interpretation of painful body regions. How we defined areas on the pain drawing may be substantially different from how these locations were interpreted by subjects completing the troublesomeness grid. Additionally shading a painful area and indicating that this is troublesome may be different constructs especially at the lower end of the Likert scale for troublesomeness [15]; our analysis of the measure's construct validity lends some support to this notion. Thus, notwithstanding these item non-completion rates we conclude that the grid has acceptable face validity.

### Criterion and construct validity

The grid behaves as expected with our other measures, correlating well with the pain intensity components of the CPG, There is an inverse correlation with the EQ 5D indicating that the more troublesome the pain, the greater the negative effect on quality of life. The correlation between troublesomeness and psychological distress was weaker than the correlation with more physical measures. For all of these comparisons the strength of the correlations will be weakened by the different time frames used; CPG six months, troublesomeness four weeks, GHQ 12 and EQ 5D today. It is impossible not to use these different time frames as all of the health outcome measures have been validated to measure the impact of pain over these particular time periods, if we had changed the wording of the measures so that they all measured pain over the same time period, this may have affected their validity. Taking this into consideration, and also the fact that we are comparing a single item question on one body region with overall measures of health status, these results indicate that the troublesomeness grid is behaving in a predictable manner. The multivariate analysis indicates that pain intensity was the strongest explanatory variable, followed by health related quality of life, but that even including all our variables plus age and sex explains less than 40% of the variance in the data. We may have been able to explain more than 40% of the variance in the data if we had included other outcome measures within our questionnaire. For example, one could hypothesize that fear avoidance behaviour and functional ability may be related to troublesomeness. One might expect that patients who experience their pain as troublesome may be more likely to adopt fear avoidance behaviours and that in turn this may lead to functional impairments. One could also hypothesize that the existence of coexisting medical conditions may help to explain troublesomeness of pain, as those with coexisting medical conditions may place a lower priority on the troublesomeness of their pain relative to the troublesomeness of their coexisting medical condition.

However, our analysis in this study suggested that troublesomeness is related to both pain intensity and health related quality of life but not psychological distress.

### Reliability

The retest questionnaires were completed one month after the initial survey. During this time there will inevitably be some change in troublesomeness. In spite of this we obtained good levels of exact agreement and even better levels of agreement for at least moderately troublesome pain suggesting that the measure is sufficiently reliable for our needs. The intra-class correlation coefficients also demonstrated that there was a high level of agreement between test and retest which further adds to our confidence in this measure.

The results show that this approach to collecting simple data on the impact of pain in different regions appears to be suitable for use in a postal questionnaire. Our primary purpose was not to develop a new overall pain measure, as many such are already available. However, many of those with chronic pain have pain in multiple regions and the troublesomeness grid is one way of distinguishing the impact of pain in different regions. This approach can enable a comparison of for example the impact of generic treatments on different parts of an individuals' overall pain. For example, using this as an outcome measure in a study of exercise treatment for low back pain would provide some information on exercises' effect on other painful areas, without greatly increasing the participants' questionnaire burden.

## Conclusion

Based on these findings and the previous validation of similar questions when just used as two items on back and leg pain, we can conclude that we can use the individual components of the troublesomeness grid to compare the impact of pains in different regions using identically worded questions. We will do more detailed analysis of the relationship between these different painful regions and their health impact in future studies.

## Abbreviations

GHQ 12 – General Health Questionnaire 12

CPG – Chronic Pain Grade Questionnaire

MSPQ – Modified Somatic Perceptions Questionnaire

EQ 5D – EuroQol Health Related Quality of Life questionnaire

## Competing interests

The author(s) declare that they have no competing interests.

## Authors' contributions

SP, MU, and DC designed this study and developed the analysis plan. SP and MU wrote the first draft of the paper. SP managed the baseline pilot and full baseline epidemiological survey and did the analysis for the paper along with DC. DC commented on all drafts of the paper and ran the follow up survey. MU commented on subsequent drafts of the paper and was the principal investigator for both the baseline survey and its follow up. AB, TP, SV, NF designed the BEXS study which included the baseline survey from which data was used in this paper, they also commented on successive drafts of this paper.

## Pre-publication history

The pre-publication history for this paper can be accessed here:



## Figures and Tables

**Table one:** Descriptive statistics of study health outcome measures – main study

	Patients with chronic pain	Whole sample
Mean EQ 5D (SD)	0.63 (0.26)	0.79 (0.24)
Range	(-0.35,1.00)	(-0.35, 1.00)
Mean GHQ 12 score (SD)	13 (5.6)	11.8 (5.2)
Range	(2, 36)	(0,36)
Mean pain intensity	54 (20.9)	32.4 (28.4)
Range	(0–100)	(0–100)
Mean pain related disability	37 (29.6)	20.7 (27.4)
Range	(0–100)	(0–100)

**Table two:** At least moderately troublesome pain and pain intensity, disability, health related quality of life and psychological distress

Body region	Pain intensity* CPG	Disability* CPG	EQ 5D*	GHQ 12 ^†^
Lower back	0.34 P = 0.002^‡^	0.29 P = 0.009	-0.25 P = 0.02	0.26 P = 0.00
Knee	0.33 P = 0.003	0.25 P = 0.02	-0.25 P = 0.02	0.17 P = 0.00
Neck	0.30 P = 0.007	0.34 P = 0.002	-0.24 P = 0.036	0.19 P = 0.00
Shoulder	0.20 P = 0.007	0.25 P = 0.02	-0.42 P = 0.00	0.18 P = 0.00
Hip/thigh	0.34 P = 0.002	0.39 P = 0.0200	-0.19 P = 0.00	0.20 P = 0.00

* Pilot study data n = 201, ^† ^main study data n = 1979 ^‡ ^Spearman's rho correlation coefficients

**Table three:** Factors associated with troublesome pain in the low back, shoulder, knee, neck and hip/thigh

	Factors associated	OR	**CI**		**R**^2^
			Lower	Upper	
**Low back pain**	***Age***	0.99	0.99	1.01	**0.37**
	***Gender***	0.89	0.69	1.14	
	***Education***	1.07	0.83	1.39	
	***Work***	1.28	0.95	1.74	
	***EQ5D***	0.33*	0.17	0.68	
	***GHQ 12***	1.01	0.99	1.04	
	***Pain intensity***	1.04*	1.03	1.05	
	***Disability***	***1.00***	***0.99***	1.01	
**Shoulder**	***Age***	1.01	0.99	1.02	**0.28**
	***Gender***	0.63*	0.48	0.83	
	***Education***	0.99	0.75	1.32	
	***Work***	1.21	0.88	1.68	
	***EQ5D***	1.08	0.55	2.12	
	***GHQ 12***	1.00	0.98	1.03	
	***Pain intensity***	1.05*	1.04	1.05	
	***Disability***	***1.00***	***0.99***	1.01	
**Neck**	***Age***	1.00	0.99	1.01	**0.25**
	***Gender***	0.67*	0.51	0.86	
	***Education***	0.84	0.64	1.11	
	***Work***	1.25	0.90	1.73	
	***EQ5D***	0.74	0.38	1.44	
	***GHQ 12***	1.01	0.99	1.04	
	***Pain intensity***	1.04*	1.03	1.05	
	***Disability***	***1.00***	***0.99***	1.01	
**Hip/thigh**	***Age***	1.03*	1.01	1.04	**0.33**
	***Gender***	0.67*	0.50	0.90	
	***Education***	0.85	0.62	1.16	
	***Work***	1.16	0.81	1.67	
	***EQ5D***	0.27*	0.14	0.56	
	***GHQ 12***	1.01	0.98	1.04	
	***Pain intensity***	1.03*	1.03	1.04	
	***Disability***	0. 45*	***0.97***	0.12	
**Knee**	**Age**	1.02*	*1.01*	*1.03*	**0.26**
	**Gender**	*0.92*	*0.70*	*1.19*	
	**Education**	*0.95*	*0.72*	*1.26*	
	**Work**	*1.27*	*0.91*	*1.77*	
	**EQ5D**	0.35*	*0.18*	*0.69*	
	**GHQ 12**	*0.99*	*0.97*	*1.02*	
	**Pain intensity**	1.03*	*1.02*	*1.04*	
	**Disability**	**1.00**	**0.97**	*1.01*	

*Represents significant results at the 5% level.

**Table four:** Test-retest reliability of the troublesomeness grid N = 30

**a) Percentage agreement**			
	A No troublesome pain	B Close agreement	A+B Overall agreement

Neck	14 (47%)	11 (37%)	25 (83%)
Shoulders	14 (47%)	7 (23%)	21 (70%)
Low back	15 (50%)	11 (37%)	26 (87%)
Hip/thigh	18 (60%)	9 (30%)	27 (90%)
Knee	11 (37%)	14 (47%)	25 (83%)
**b) Intraclass correlation coefficients**			

	Intra-class correlation coefficient	95% Confidence Interval	

Neck	0.80	0.57, 0.91	
Shoulders	0.59	0.11,0.81	
Low back	0.89	0.76, 0.95	
Hip/thigh	0.91	0.80,0.96	
Knee	0.91	0.81,0.96	

A:- Those with no pain or not at all troublesome pain in both surveys

B:-Those with at least moderately troublesome pain in either survey who remained the same or changed by one Likert point between surveys

A+B:- Overall agreement between baseline and follow up
